# Detection of Brevetoxin in Human Plasma by ELISA

**DOI:** 10.1093/jat/bkab010

**Published:** 2022-03-21

**Authors:** Brady R. Cunningham, Rebecca M. Coleman, Adam M. Schaefer, Elizabeth I. Hamelin, Rudolph C. Johnson

**Affiliations:** 1 Division of Laboratory Sciences, National Center for Environmental Health, Centers for Disease Control and Prevention, Atlanta, GA 30341, USA; 2 Harbor Branch Oceanographic Institute, Florida Atlantic University, Ft. Pierce, FL 34946, USA

## Abstract

Florida red tides have become more common and persistent in and around the Gulf of Mexico. When in bloom, red tides can produce brevetoxins in high concentrations, leading to human exposures primarily through contaminated food and ocean spray. The research described here includes adapting and validating a commercial brevetoxin water test kit for human plasma testing. Pooled plasma was fortified with a model brevetoxin, brevetoxin 3, at concentrations from 0.00500 to 3.00 ng/mL to generate calibration curves and quality control samples. The quantitative detection range was determined to be 0.0400–2.00 ng/mL brevetoxin 3 equivalents with inter- and intraday accuracies ranging from 94.0% to 109% and relative standard deviations <20%, which is within the US Food and Drug Administration guidelines for receptor-binding assays. Additionally, cross-reactivity was tested using 4 of the 10 known brevetoxins and 12 paralytic shellfish toxins. The cross-reactivity varied from 0.173% to 144% for the commercially available brevetoxin standards and 0% for the commercially available paralytic shellfish toxin standards. Fifty individual unexposed human plasma samples were measured to determine the limit of detection and endogenous interferences to the test. The validated method was used to test 31 plasma samples collected from humans potentially exposed to brevetoxins, detecting 11 positives. This method has been proven useful to measure human exposure to brevetoxins and can be applied to future exposure events.

## Introduction

Brevetoxins (PbTxs) are lipid-soluble, cyclic polyether neurotoxins produced by the dinoflagellate *Karenia brevis. K. brevis* naturally occurs in the Gulf of Mexico and the Caribbean Sea with additional reports of suspected blooms in Australia, New Zealand and Japan (1). In the USA, Florida red tides are attributed to *K. brevis* blooms. During bloom events, PbTxs may be produced in high concentrations, released into the water and concentrated in shellfish or aerosolized via sea spray. PbTxs are not toxic to shellfish, but are a neurotoxin affecting humans, marine mammals, fish and birds. Shellfish concentrate PbTxs during filter feeding and become a vector of PbTx exposure when consumed.

When PbTx enters the body, the toxin binds to site 5 of the voltage-gated Na channel inducing a channel-mediated Na+ ion influx leading to neurotoxic shellfish poisoning (NSP) (1, 2). Similar to a mild case of paralytic shellfish poisoning (PSP) (2), NSP commonly causes gastrointestinal distress and, in rare cases, paralysis, seizures and coma (3–5). Symptoms from PbTx ingestion begin 3–18 h after exposure and may last several days (5). Another route of PbTx exposure is inhalation, which produces less severe symptoms such as respiratory irritation and bronchoconstriction. Symptoms following PbTx inhalation begin minutes to hours (<24 h) after exposure and last 2–3 days, with no reported long-term or chronic effects (2). PbTx-exposure experiments on sheep have found evidence for hyper-responsiveness and lung inflammation following repeated and chronic exposure to PbTx-3 aerosols (6). Those results indicate the need for further study into long-term or chronic effects of PbTx inhalation exposure in humans.

PbTxs can be categorized into two structural groups based on the molecular backbone, forming the parent molecules Brevetoxin A (10 rings) and Brevetoxin B (11 rings) (1, 7–9). Presently, >10 PbTx analogs were isolated and identified, a majority of which have the Brevetoxin B backbone (10, 11). PbTx-3 and PbTx-2 contain the Brevetoxin B backbone and are the most common analogs found in water and aerosols (12). Toxicity differs both between analogs and methods of exposure (13). When PbTx-3 and PbTx-2 were administered to mice via intraperitoneal injection, the potencies were similar, but when administered orally, PbTx-3 was 10-fold more potent than PbTx-2 (LD_50_ (median lethal dose) value of 520 mg/kg bodyweight for PbTx-3 and 66,000 mg/kg bodyweight for PbTx-2) (14, 15). The difference in toxicity may relate to absorption rate of the analogs (9, 16). For humans, it is estimated that an exposure of 2–3 μg PbTx-3 equivalents/kg bodyweight is necessary to produce toxic effects and total body clearance can take up to 6 days (17). The resulting NSP is not fatal, but there is no known antidote and treatment consists of supportive care (2).

Various cases of NSP or PbTx exposure have been reported in the scientific and medical literature. A majority of these studies measured PbTx exposure in marine and terrestrial animals (e.g., 6, 11, 18–24), with a comparatively smaller amount focusing on PbTxs in humans. Reports of human PbTx exposure from ingestion are extremely rare. One of the cases that attracted much public attention occurred in Sarasota Bay, Florida, in 1996. Several family members were admitted to a hospital presenting NSP symptoms after eating shellfish. Ultimately, the diagnosis was confirmed using radioimmunoassay and receptor-binding assay to detect brevetoxin in urine (5).

More commonly, humans are exposed to aerosolized PbTxs via sea spray and report respiratory irritation symptoms (12, 25–30). These exposures were confirmed using a variety of methods and instruments, some specific to PbTx (high-performance liquid chromatography mass spectrometry (HPLC–MS) and enzyme-linked immunosorbent assay (ELISA)) and others non-specific to PbTx (cell staining and lung-function tests). However, there is no clinical method to detect PbTx in human plasma. Here, we evaluate the suitability of a commercially available ELISA kit to accurately and precisely quantitate PbTx-3 in human plasma. This kit was originally intended to quantitate PbTx-3 in seawater and shellfish tissue extract. We confirmed that this ELISA kit can accurately quantitate PbTx-3 in human plasma from 0.0400 to 2.00 ng/mL with varying cross-reactivity to other common PbTx analogs.

## Methods

### Materials

Commercial standards of brevetoxin 3 (100 μg) and brevetoxin 2 (100 μg) were purchased from Abcam Inc. (Boston, MA). Brevetoxin 1 (100 μg) and brevetoxin B5 (100 μg) were purchased from MARBIONC (Wilmington, NC). Brevetoxin 9 (100 μg) was purchased from Accurate Chemical and Scientific Corp (Westbury, NY). All materials were stored in glass vials at −20°C. The ELISA kit (part number 520026) was purchased from Eurofins Abraxis (Warminster, PA). During the initial phase of method validation (July 2019), only one commercial brevetoxin ELISA kit was available for purchase. Protein LoBind tubes (2 mL) were purchased from Eppendorf (Hauppauge, NY). Pooled and individual plasmas were purchased from Tennessee Blood Services (Memphis, TN). Plasma samples were acquired from anonymous, random individuals, representative of the general population, and thus, this work did not meet the definition of human subjects research as specified in 45-CFR 46.102 (f).

### Preparation of calibrators, quality control samples, anchors and quality materials

For validation, calibrators were prepared at 0.0400, 0.0600, 0.200, 0.220, 0.500 and 2.00 ng/mL PbTx-3, quality control (QC) samples were prepared at 0.100 and 1.00 ng/mL PbTx-3, and anchors were prepared at 0.00500 and 3.00 ng/mL PbTx-3. QC samples were used to ensure the accuracy of the calibration curve. Quality materials were used to determine accuracy through sample fortification, precision and stability. These materials consisted of two pools: pool 1 was fortified at 0.0500, 0.150 and 0.200 ng/mL PbTx-3, and pool 2 was fortified at 0.100, 0.250 and 0.500 ng/mL PbTx-3. Calibrators, QCs, anchors and quality materials were prepared in glass volumetric flasks at the beginning of the validation, transferred into 2-mL protein LoBind tubes at 550 μL volumes for multiuse aliquots, frozen at −20°C and thawed as needed. Ten individual plasmas were fortified with PbTx-3 at two levels, 0.100 and 0.220 ng/mL.

### Brevetoxin ELISA

All reagents within the kit were brought to room temperature, before beginning the ELISA procedure. Samples (QCs, calibrators, blank plasma, quality materials and unknowns) were centrifuged for 5 min at 10,000 × g to pellet particulate that may interfere with a competitive ELISA. Thirty microliters of phosphate-buffered saline followed by 20 μL of each sample and 50 μL of polyclonal sheep antibody solution were added to each well of the plate with a multichannel pipet. The ELISA plate was covered with an adhesive seal and placed on a ThermoMixer C (Hauppauge, NY) for 1 h with intervals of 30 s of shaking at 800 revolutions per minute (RPM), followed by 2 min without shaking. The plate was removed from the shaker and contents were removed by dumping them into the waste via vigorous shaking, and the wells were washed 3 times with 250 μL wash buffer. One hundred microliters of color substrate solution were added to each well of the plate. The plate was then covered again with an adhesive seal and placed back on a ThermoMixer C for 30 min with intervals of 30 s of shaking at 800 RPM, followed by 2 min of no shaking. After this incubation, 100 μL of stop solution was added to each well to prevent further color development. The plate was immediately transferred to a BioTek PowerWave HT microplate spectrophotometer (Winooski, VT), and absorbance was read at 450 nm. Absorbance values for each calibrator were normalized to the pooled plasma blank. The normalized values were plotted against theoretical concentrations and fit to a four-parameter fit curve using BioTek Gen5 software (v2.04).

### Cross-reactivity

Calibration curves for brevetoxin commercial standards (PbTx-1, PbTx-2, PbTx-B5 and PbTx-9) and PSP commercial standards (saxitoxin, neosaxitoxin, decarbamoyl-saxitoxin, gonyautoxins-1/4, gonyautoxins-2/3, decarbamoyl-gonyautoxin-2, decarbamoyl-gonyautoxin-3, *N*-sulfocarbamoyl toxins C-1/2 and domoic acid) were prepared from 0.00100 to 1,000 ng/mL and analyzed in duplicate according to the ELISA protocol above. The IC_50_ from each curve were used to calculate cross-reactivity of each analyte with respect to PbTx-3 (percent cross-reactivity = (IC_50_ (half-maximal inhibitory concentration) PbTx-3/IC_50_ Analyte) × 100) (31).

## Results

### Validation of PbTx-3 in human plasma

We evaluated the proficiency of this ELISA kit to quantify PbTx-3 in human plasma. The quantitative range of PbTx-3 in plasma was tested from 0.00500 to 3.00 ng/mL, and the optimal range was determined to be 0.0400 to 2.00 ng/mL. Interday accuracy and precision of calibrators and QCs were measured by evaluating 20 cumulative curve measurements and calculating percent accuracy and relative standard deviation (RSD). Calibrators and QCs were analyzed over the course of 8 weeks by two analysts using two kit lots (19G0154 and 19G0321), with no more than two sets of calibrators and QCs per day. Interday percent accuracies for the 20 calibrator and QC replicates ranged from 98.0 to 106% and the RSD ranged from 10.1 to 19.0% (Table I). The use of anchor points at 0.00500 and 3.00 ng/mL significantly improved the accuracies at the high and low end of the curve and kept the R^2^ value above 0.98. These results fall within the criteria outlined by the US Food and Drug Administration (FDA) for ligand-binding assays (LBAs) (32).

**Figure 1.**
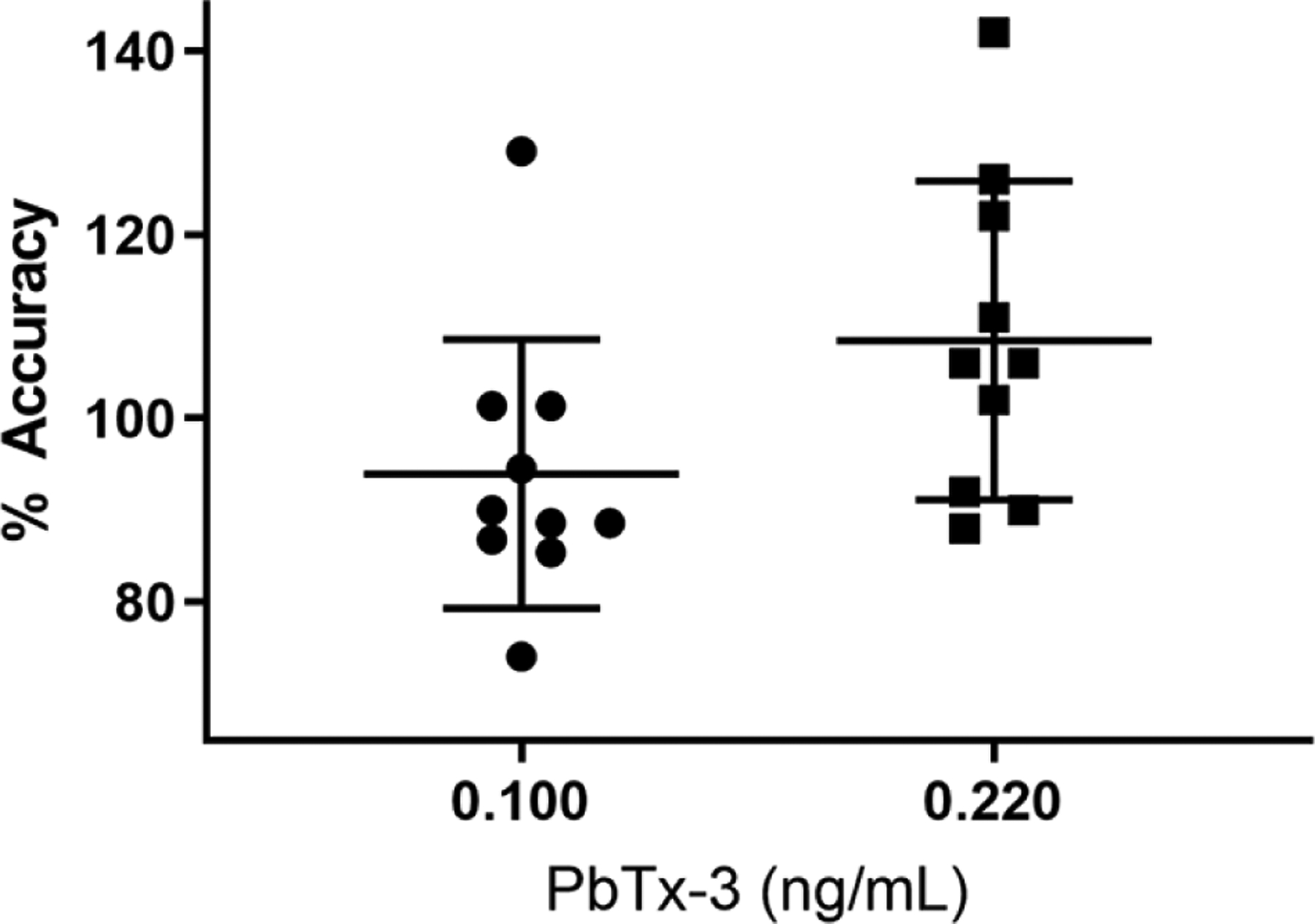
Percent intraday accuracies of individual plasmas (*n* = 10) fortified at 0.100 and 0.220 ng/mL PbTx-3.

We tested the method performance by measuring 50 individual blank plasmas for interferences and fortifying 10 individual human plasmas with 0.100 and 0.220 ng/mL PbTx-3 equivalents to ensure method accuracy in among individual samples. The 50 individual blanks all measured <50% of the lowest calibrator (0.04 ng/mL PbTx-3 equivalents) (data not shown). The average intraday accuracy of the fortified individuals was 109% for the 0.100 ng/mL and 94.0% for the 0.220 ng/mL fortified individual plasmas. The RSD was 16.0% for the 0.100 ng/mL and 15.6% for the 0.220 ng/mL fortified individual plasmas (Figure 1).

### Method ruggedness

To assess the ruggedness of this method, additional experiments were performed to determine recovery, quality material precision and stability. Recovery was measured using two different pools of plasma, fortified at three levels. Pool 1 recovery ranged from 87.6 to 94.0%, and pool 2 recovery ranged from 104 to 119%. The mean recovery for both pools was 101% with 11.8% RSD. Precision was determined by calculating the RSD across 20 total runs and within 2 runs per day for each QC material (0.220 and 0.100 ng/mL). Precision for quality material 1 (0.220 ng/mL) was 11.5%, and quality material 2 (0.100 ng/mL) was 16.5%. Stability was assessed after initially measuring each quality material and then measured again after the material went through four separate processes; (i) 30 days stored at −20°C to determine long-term stability, (ii) left at room temperature overnight to assess short-term, bench-top stability, (iii) remeasured processes samples after sitting overnight and (iv) three freeze-thaw cycles to mimic sample handling conditions (Table II).

### Cross-reactivity

**Figure 2.**
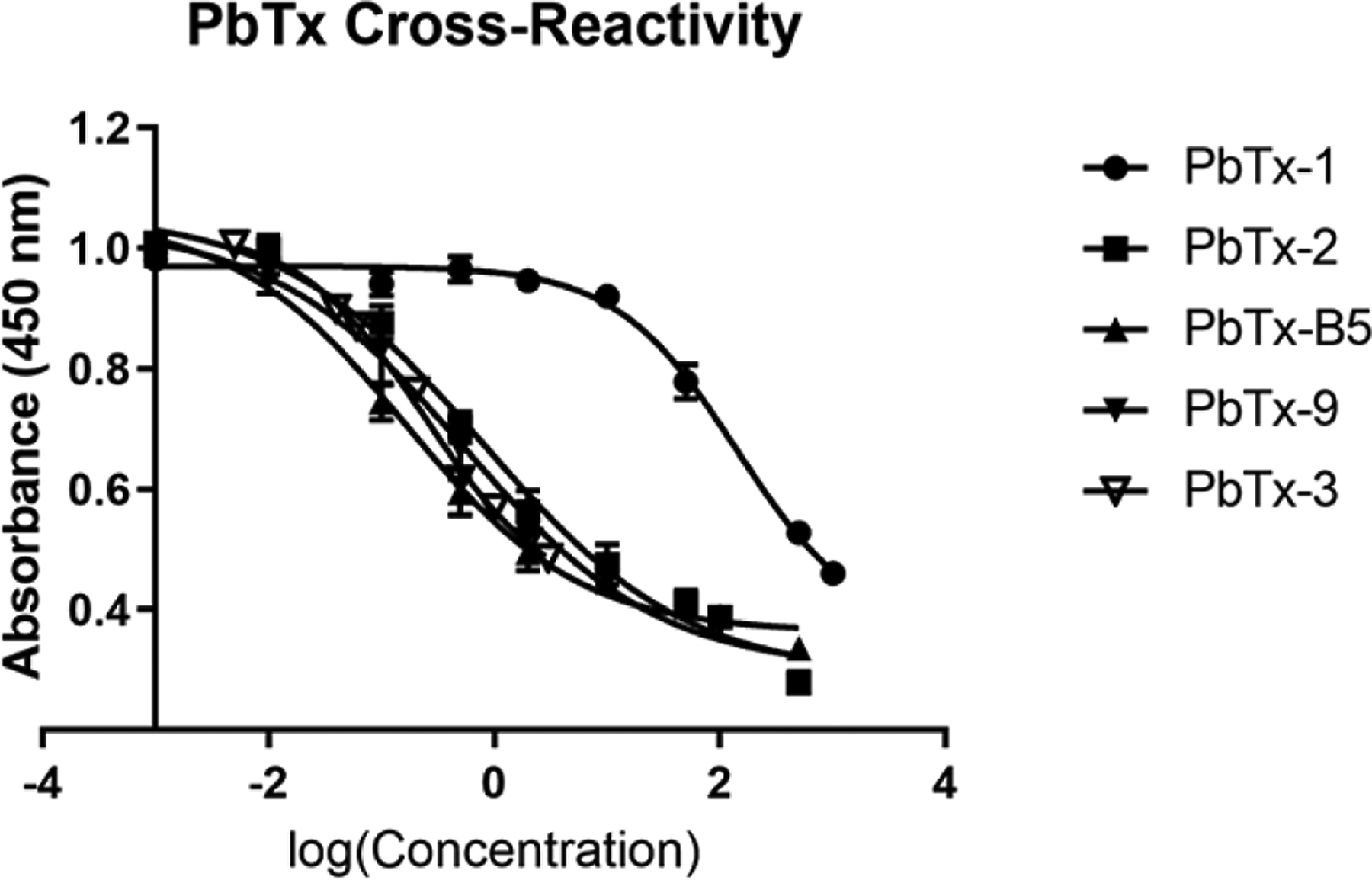
Comparison of cross-reactivities for various brevetoxin analytes ranging from 0.00100 to 1000 ng/mL per analyte.

We found that this ELISA cross-reacts 0.173% with PbTx-1, 29.6% with PbTx-2, 144% with PbTx-B5, and 44.1% with PbTx-9 (Figure 2). There was no cross-reactivity with any of the PSP commercial standards.

### Plasma sample analysis from florida residents

During the summer and fall of 2018, the state of Florida experienced multiple harmful algal blooms including cyanobacteria and *K. brevis*. As a part of a statewide human exposure assessment, urine, nasal swabs and blood were collected from residents of bloom-affected regions including the city of Fort Myers. The cross-sectional study included a comprehensive questionnaire, which gathered information on symptomology and medical history as well as potential route, duration and location of exposure at the time of sample collection. A total of 69 individuals were screened in the Fort Myers area during October of 2018. Of those individuals, 31 were selected that had potential exposure to PbTx based on the survey responses and provided blood samples with sufficient volume and quality for analysis using the method described in this paper.

**Figure 3.**
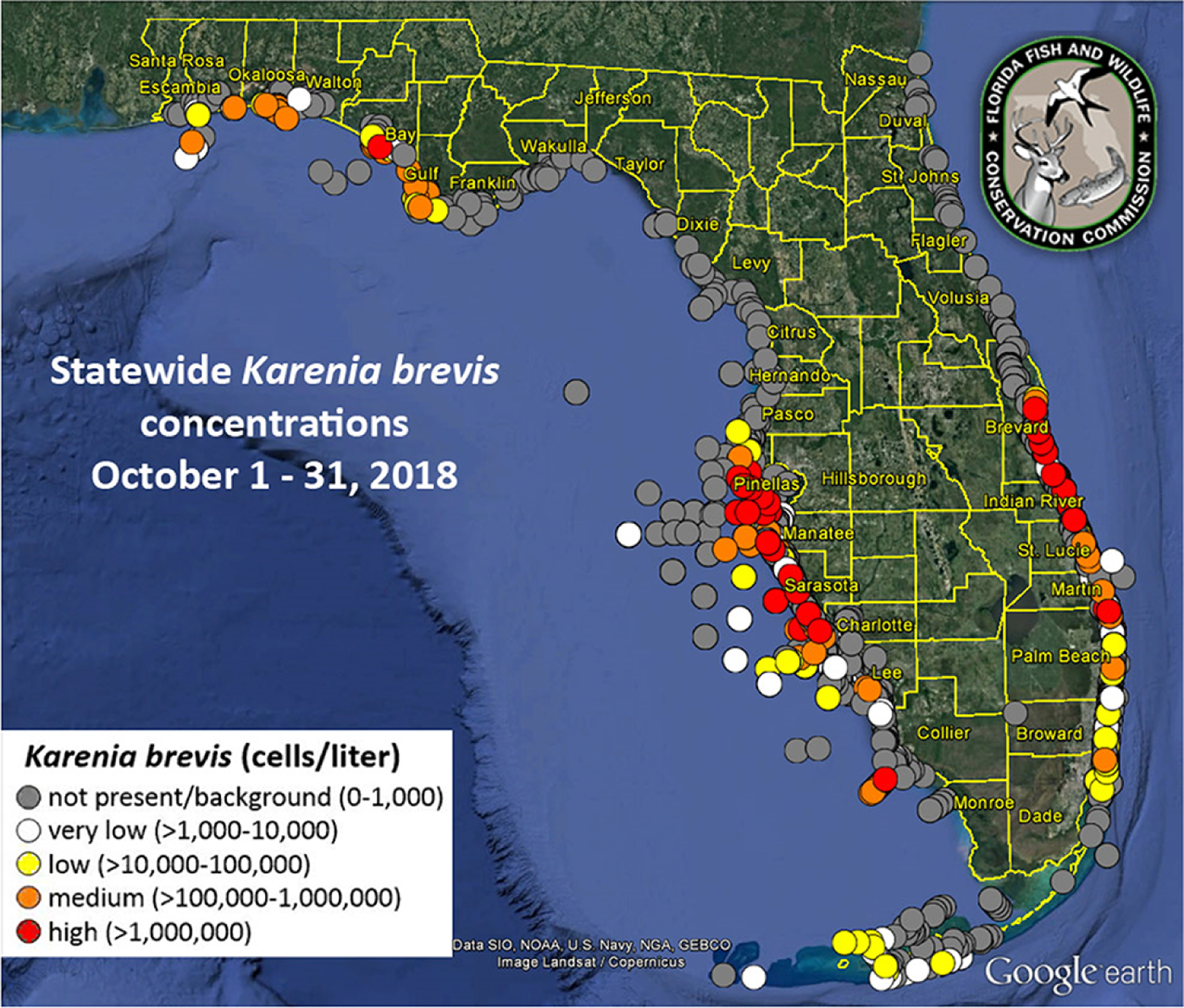
*Karenia brevis* cell concentrations during exposure. Photo courtesy of Florida Fish and Wildlife Conservation Commission (40).

Concurrent with the human subject recruitment, multiple agencies collected water and algae samples for bloom and toxin surveillance. Between July and November, surface water concentrations of *K. brevis* ranged from below detection to 110 g/L, with one of the largest blooms occurring in October 2018 (Figure 3). More specific geographical and spatial-temporal environmental sampling and human exposure data are currently being analyzed and will be reported elsewhere.

Eleven of the 31 plasma specimens analyzed by the ELISA method described in this paper yielded positive results (Table III). Of the individuals with positive results, 64% reported exposure or contact with the waters from the Gulf of Mexico or intercostal waterways within the 30 days prior to sample collection. The individual with the highest reported concentration of 0.421 ng/mL reported a residence directly on a local waterway.

## Discussion

Florida red tides have affected humans since first documented in 1844 (3, 33). However, there is little published data on PbTx concentrations in human clinical samples. One of the most discussed studies reported a range of 42–117 ng/mL total PbTx in urine collected from two individuals presenting NSP symptoms after ingestion of contaminated shellfish (5). Diagnosis of NSP from PbTx ingestion is rare and maybe underreported, but the most commonly reported route of exposure for PbTX is inhalation (2, 12). Since the amount of PbTx inhaled is anticipated to be significantly less than the amount ingested from contaminated shellfish, sensitive methods are needed to measure PbTx in clinical specimens. Our method was designed to detect sub-ng/mL concentrations, reporting PbTx-3 equivalents in plasma as low as 0.0400 ng/mL to support both ingestion and inhalation studies. The reportable limit and specificity were confirmed by the analysis of 50 individual plasma samples with no known exposure to PbTx to assure that all positive results are indeed from exposure and not interferences.

Several studies indicate that chronic, low-level exposure of aerosolized PbTx could lead to acute, subacute and possibly other chronic illnesses for asthmatics and others with respiratory illnesses (6, 12, 34–36). Additionally, neuronal degeneration was also found in mice exposed to aerosolized PbTx-3 has been reported, which may indicate potential negative effects for humans with existing neurodegenerative diseases (21). Future epidemiological studies may be helpful in determining the population impacts of these toxins from inhalation exposures. This would require a method with high accuracy and precision for data comparison across time and between studies to screen potential PbTx exposures. Our method met these needs with QC accuracy and precision ranging from 101 to 106% and 17.7 to 19.1%, respectively, values which are within the FDA requirements for LBAs. Also, individual plasmas fortified with PbTx-3 at 0.100 and 0.220 ng/mL were assessed and confirmed the ability of this method to provide accurate results (average accuracy of 109 and 94.0%, respectively) between individual persons and demonstrates the ruggedness of the method for use in epidemiological studies.

During red tides, humans may be exposed to multiple PbTx analogs. This method has demonstrated cross-reactivity with multiple Brevetoxin B backbone analogs. According to the data sheet from the ELISA kit, this assay cross-reacts 133% with deoxy PbTx-2, 127% with PbTx-5, 102% with PbTx-2, 83% with PbTx-9, 13% with PbTx-6 and 5% with PbTx-1. During our testing, we found that this ELISA cross-reacts 29.6% with PbTx-2, 44.1% with PbTx-9, 0.173% with PbTx-1 and 144% with PbTx-B5. We did not test the cross-reactivity of deoxy PbTx-2, PbTx-5 and PbTx-6 due to the unavailability of commercial standards at the time of data collection. This variation in cross-reactivities between the kit insert and our data may be due to matrix difference, as the kit was originally designed for saltwater and shellfish samples and our method used human plasma. Also, this kit uses polyclonal antibodies, which can vary between lots. For use in exposure assessments, it would be ideal to capture and detect all PbTx analogs, but this method should be able to detect the majority of PbTx exposures since PbTx-3 and PbTx-2 are the most common analogs and this method was validated using PbTx-3 and had a 44.1% cross-reactivity with PbTx-2 (13, 37). Finally, a majority of PbTx analogs and cysteine adducts share the same Brevetoxin B backbone as PbTx-3 and PbTx-2, which should also increase the chance for PbTx detection (1, 38, 39).

Here, we demonstrated the ability of this method to detect low-dose human exposures to PbTxs from study participants living or working near red tides in Florida. During red tide events, contaminated air in proximity to the blooms mainly contains PbTx-3 and PbTx-2 (6, 12, 24, 36). The analysis of plasma samples from this study confirmed the application of this method for inhalation studies and revealed a range of 0.0430–0.421 ng/mL PbTx-3 equivalents for the 11 of 31 positive samples. Study participants’ questionnaire responses indicated varying levels of contact with contaminated water from none to direct contact. The interpretation of those data will be included in a larger harmful algal bloom data set and discussed and reported elsewhere. Further studies examining the potential long-term health effects associated with chronic PbTx exposure may yield substantive findings. Overall, these results further reinforce the need for clinical methods detecting PbTx in humans.

## Conclusion

In conclusion, we describe a sensitive and simple quantitative ELISA in human plasma that can complement PbTx monitoring programs and provide valuable data on human exposure. These tests are cost-effective and do not require expensive equipment and extensive training to perform the assay. Furthermore, this method should detect a majority of PbTx analogs with varying toxicities due to the antibody cross-reacting with other Brevetoxin B backbone analogs. Ideally, the results from this method would be confirmed with a more targeted approach like HPLC-MS to discern which specific PbTx analogs are present. However, confirmatory method development has remained a challenge due to required sensitivity, biological matrix effects and the limited availability of PbTx commercial standards including metabolized PbTx cystine adducts (38). Overall, this manuscript highlights the need for further and more comprehensive investigation of PbTx exposure from the public health standpoint, as well as the need for confirmatory analysis to identify which PbTx analogs are present within the samples.

## Figures and Tables

**Table I.** Results from Method Validation. Interday (*n* = 20) Percent Accuracy and RSD for Calibrators and QCs

	Brevetoxin 3 (ng/mL)	Average (ng/mL)	Percent accuracy	Percent RSD
Calibrators	0.0400	0.0392	98.0	14.4
	0.0600	0.0640	106	13.0
	0.200	0.197	98.4	10.7
	0.220	0.217	98.4	10.1
	0.500	0.516	103	13.0
	2.00	2.09	104	13.9
QCs	0.100	0.101	101	17.7
	1.00	1.07	106	19.1

**Table II.** Quality Material Stability Assessment. Average (*n* = 3) Percent Difference from Initial Measurement of Quality Materials (*n* = 3)

Initial measurement (ng/mL)	Percent difference from initial measurement			
	Long-term stability	Bench top stability	Processed sample stability	Three freeze-thaw cycles
0.117	+9.90	−8.20	−13.6	−7.40
0.223	+ 16.7	+3.40	−4.00	−2.20

**Table III.** Positive ELISA Results of Plasma Samples from Florida Study Participants and Summarized Method Validation

Positive individuals	Plasma analysis (ng/mL PbTx-3 equivalents)
1	0.179
2	0.0610
3	0.0620
4	0.421
5	0.236
6	0.0540
7	0.0580
8	0.0540
9	0.0430
10	0.0590
11	0.0710

Method limit of detection: 0.0400 ng/mL PbTx-3 equivalents.

Method Precision: 10.1–19.1%.

Method Accuracy: 94–109%.
